# Building an implementation framework to address unmet contraceptive care needs in a carceral setting: a systematic review

**DOI:** 10.1186/s40352-023-00243-8

**Published:** 2023-10-20

**Authors:** Douglas Routh, Rebecca Simmons, Jessica Sanders, Alexandra Gero, Hannah Aanderud Tanner, David K. Turok

**Affiliations:** Division of Family Planning, Department of Obstetrics & Gynecology, University of Utah School of Medicine, 30 N 1900 E, Room 2B200, Salt Lake City, UT 84132 USA

**Keywords:** Contraception, Incarcerated sexual and reproductive health care, Policy recommendations, Systematic review, Implementation science

## Abstract

**Background:**

The provision of contraceptive care for incarcerated individuals has been largely inconsistent and has contributed to, at best, inadequate care, and at worst reproductive abuses, violence, and coercion. While previous research has identified strategies to remedy known issues, to date, very few recommendations have been implemented across the carceral system. To address this, we conducted a systematic review of policy and practice recommendations to improve contraceptive care to reproductive-aged, incarcerated individuals in the United States.

**Methods:**

We conducted this systematic review utilizing the Joanna Briggs Institute methodology and framed it within the National Implementation Research Network’s (NIRN) Exploration stage. We searched PubMed, PSYCInfo, SCOPUS, ProQuest, Web of Science, MedLine, Social Science Citation Index and reference sections of included materials. Basic study information, explicitly stated policy and practice recommendations, and discussions and conclusions that subtly provide recommendations were extracted in full text. We utilized a thematic analysis approach to analyze the extracted text.

**Results:**

A total of 45 materials met the inclusion criteria. Seven overarching themes were identified: 1) policy changes needed to implement care; 2) need for contraceptive care in carceral systems; 3) justice agency barriers regarding contraceptive care provision; 4) policy barriers to contraceptive access; 5) funding strategies to improve care; 6) patient preferences for contraceptive care delivery; and 7) healthcare provider knowledge regarding contraceptive care. The seven themes identified shed light on the need for, gaps, barriers, and facilitators of current contraceptive care provision to incarcerated individuals.

**Conclusion:**

This systematic review accomplished two goals of NIRN’s Exploration stage. First, the compiled evidence identified a clear need for change regarding policies and practices pertaining to contraceptive care provision to incarcerated individuals in the United States. Second, our findings identified several evidence-based solutions supported both by research and professional healthcare organizations to address the identified need for change. This study provides an initial blueprint for correctional agencies to implement the necessary changes for improving contraceptive care provision to incarcerated populations. The correctional system is in a unique position to deliver much-needed care, which would result in many potential benefits to the individuals, correctional system, and community at large.

**Supplementary Information:**

The online version contains supplementary material available at 10.1186/s40352-023-00243-8.

Over the past three decades, research conducted in the United States has supported the provision of contraception services to women^1^ including devices, prescriptions, education, and connections to community health centers during incarceration and prior to or at release (see Clarke et al. 2006a*; Clarke et al. 2006b*; Clarke et al. 2006c*; Knittel, 2019 ; Knittel et al. 2017*; Peart & Knittel, 2020*; Schonberg et al. 2015*; Shlafer et al. 2019 ; Sufrin et al. 2014*; Sufrin et al. 2019; Sufrin et al. 2009a*, 2009b for examples). Professional healthcare organizations such as the American Congress of Obstetricians and Gynecologists (ACOG, 2012*, 2021), the Association of Women’s Health, Obstetric, and Neonatal Nursing (AWHONN, 2011), the American Public Health Association (APHA, 2003), and the National Commission on Correctional Health Care (NCCHC, 2014) have issued recommendations in line with these findings. Yet, care provision, access, and quality of contraceptive care in U.S. prisons and jails lags behind these recommendations. The lack of uniform standards and implementation plans across the correctional system has led to delayed, inadequate, and, at times, detrimental or denied care (Clarke et al. 2006a*; Ferszt and Clarke 2012; Franco et al. 2020; Sufrin et al. 2017*; Kasdan, 2009 ; Roth, 2004; Sufrin et al. 2009a*, 2009b).

This systematic review seeks to provide a plan for implementing the policy and practice recommendations across the correctional system identified by researchers and professional health care organizations. Specifically, we compiled those policy and practice recommendations identified for improving contraceptive or family planning services to reproductive aged incarcerated women in the United States. This review seeks to fill a gap in the literature by identifying specific strategies that can be taken by justice agencies to facilitate improvements in provision of contraception and family planning services in the correctional system.

## Methodology

In evidence-based health care (EBHC) or evidence-based medicine (EBM), the best available evidence informs policy and practice (Jordan et al. 2016; Jordan et al. 2018). We utilized a similar methodology to Ryan et al. (2018) which combines a systematic review methodology protocol with a thematic analysis of the studies included to compile evidence-based practices. This systematic review was conducted in accordance with the Joanna Briggs Institute (JBI) methodology for systematic reviews (Aromataris & Munn, 2020). Specifically, we utilized the JBI methodology to develop and conduct a thorough and methodical search of the literature with a replicable search strategy and rigorous inclusion/exclusion and study appraisal criteria. We also framed this study within the National Implementation Research Network’s (NIRN) stages of implementation; specifically, the Exploration phase (Metz, Naaom, Halle, & Bartley, 2015). This systematic review approach and implementation science framework were chosen to identify and synthesize the best available evidence from all possible information sources and ascertain the policy and practice recommendations developed by researchers and professional health care organizations for improving contraceptive and family planning services to reproductive-aged, incarcerated women in the United States.

### Search strategy

A detailed search strategy was developed with the aim of capturing both published and unpublished literatures. The comprehensive three-step search (Peters et al. 2015) consisted of: 1) an initial, limited search of PubMed and PSYCInfo, which are criminal justice, social science, medical, and health databases followed by an analysis of words in the title, abstract, and index keywords; 2) a second, full search using the same search string with all seven databases indexed in the University of Utah Library (PubMed, PSYCinfo, SCOPUS, ProQuest, Web of Science, Medline, and Social Science Citation Index), and 3) reading the reference list of each selected study to identify additional studies to include in the review. Multiple search strings were used in order to capture the different nomenclature of contraceptive and reproductive healthcare and correctional or carceral settings. The specific search strings used were: 1. contracept* AND incarcerat*, 2. contracept* use in prison OR contracept* during prison, 3. contracept* use in jail OR contracept* during jail, 4. contracept* use in carceral OR contracept* during carceral, 5. “reproduct* health” in jail, 6. “reproduct* health” in carceral, 7. “reproduct* health” during incarceration, 8. “birth control” during jail OR “birth control in jail, 9. “birth control” during prison OR “birth control in prison, 10. “birth control” during carceral OR “birth control in carceral, 11. “birth control” during incarcerat*, 12. emergency contracept* during incarcerat*, 13. emergency contracept* during prison OR emergency contracept* in prison, 14. emergency contracept* during jail OR emergency contracept* in jail, and 15. emergency contracept* during carceral OR emergency contracept* in carceral. The search was limited to studies conducted in the United States due to the differing nature of the United States’ carceral system relative to other nations. The study was further confined to studies on adult populations, and materials published in the English language. Materials were restricted by excluding studies that solely focused on a teenage or already pregnant population. Other than this restriction, materials were not restricted by study type or publication type (e.g., published, unpublished, technical report, dissertation or thesis, white paper, or null or opposite effects). The date range for the search was set to January 1, 1900 through February 28, 2022.

### Inclusion and exclusion criteria

EBHC and EBM integrate research, clinical experience and practice, and patient values thus calling for research questions that rely on these factors. Systematic reviews aiming to support EBHC and EBM must incorporate these factors in the search strategy and inclusion criteria (Richardson et al. 1995; Sackett et al. 1996; Straus et al. 2005). To ensure the incorporation of these factors, this systematic review utilized the PICo method (detailed in Table 1 below) to develop the research question, search strategy, and inclusion and exclusion criteria (see Richardson et al. 1995; Snowball, 1997; Villanueva et al. 2001).

**Table 1:** PICo criteria

**• ** Population or Problem: Population attributes or characteristics (i.e., sex, gender, race/ethnicity, or setting) or problem (i.e., illness type and severity or medical diagnosis)
**•** Phenomenon of Interest: Description of the event, intervention, service, experience, process, or policy of interest
**• ** Context: Setting, circumstances, culture, climate, environment, or other influential factors

The PICo criteria applied to this systematic review were:

- Population or Problem: Incarcerated women aged 18–44 (reproductive age) who have the ability to become pregnant
- Phenomenon of Interest: Policy and practice recommendations for improving contraceptive and family planning services from research and professional health care organizations
- Context: In the United States carceral system or setting.

A more refined set of inclusion and exclusion criteria was developed from this PICo criteria and is provided in Table 2.

**Table 2:** Inclusion/Exclusion Criteria

Inclusion Criteria	Exclusion Criteria
• Adults (18 years old and older)	• Juveniles (17 years old or younger)
• Incarcerated	• Community corrections sample only
• Focused on contraception during incarceration	• Did not focus on contraception (e.g., pregnancy care or abortion)
• United States only	• Non-U.S. sample
• Women only	• Male population
• Policy and practice recommendations made or discussed	• No policy or practice recommendations made or discussed

To be considered for inclusion, an article’s policy and practice recommendations and discussions must focus on the incarcerated population but may address continuity of care between incarceration and release into the community. The contraceptive care may be provided in either the carceral setting by a justice agency or community medical staff, or the patient may be transported to a medical facility in the community to receive care, as long as the patient received care during their period of incarceration. However, abortion access as a sole focus was excluded. Studies examining contraception care for reasons beyond pregnancy prevention, such as using contraception to treat medical issues, were also included. We included studies of women inclusive of trans and gender expansive individuals with the capacity to become pregnant, but research focused exclusively on trans and gender expansive populations was excluded.

### Study selection

Following the search using the University of Utah library system, all search results were exported via a reference manager file (.ris) which were then imported into EndNote 20 where duplicates were identified and removed. The lead author (DR) screened all citations at the title and abstract levels for relevance. Those judged relevant and those where the title and abstract reviews were inconclusive received full paper appraisal. Study information was collected initially by one reviewer (DR) and checked independently by a second reviewer (HA). Any disagreements were discussed between the two reviewers (DR and HA). A third reviewer resolved disagreements as necessary (AG). The results of study inclusion and reasons for exclusion are reported in a Preferred Reporting Items for Systematic Reviews and Meta-Analyses (PRISMA) flow diagram (Moher et al. 2009; Page et al. 2021). Asterisks in the reference section indicate which studies were included in the final selection for this systematic review.

### Study quality appraisal

Several JBI appraisal tools (Joanna Briggs Institute, 2020) were used to assess study quality. Specifically, the Randomized Controlled Trials, Quasi-Experimental, and Analytical Cross Section tools were used for quantitative studies, the Qualitative Research tool was used for qualitative studies, the Systematic Review and Research Synthesis tool for was used for scoping and systematic reviews, and the Text and Opinion tool was used for researcher and professional health care organization opinion and position statements. The quantitative and qualitative tool were used for mixed methods studies, as appropriate. Study quality was assigned to provide an additional method to examine study results within a stratification scheme to identify possible differences in outcomes or policy and practice implications within each study quality group. Lastly, study quality was only appraised for included studies. Similar to study selection, study quality appraisal was initially completed by two reviewers (DR and HA) working independently and disagreements were resolved through discussion between the two reviewers, with a third reviewer joining as necessary (AG).

### Data extraction and analysis

Basic study information (i.e., authors, publication date, title, and publication source) were extracted. Explicitly provided policy and practice recommendations, along with discussions on findings and conclusions that provided more subtle recommendations, were extracted in full text. We used a thematic analysis approach to analyze the extracted text. This analysis was conducted in three stages: 1) extracted text was coded with the codes being derived from the data using an inductive coding approach via initial and line-by-line coding; 2) similarities between codes were identified and codes were grouped together into larger overarching descriptive themes; and 3) these themes were synthesized across the studies and interpreted in relation to the research question.

## Results

### Study inclusion

**Fig. 1:**
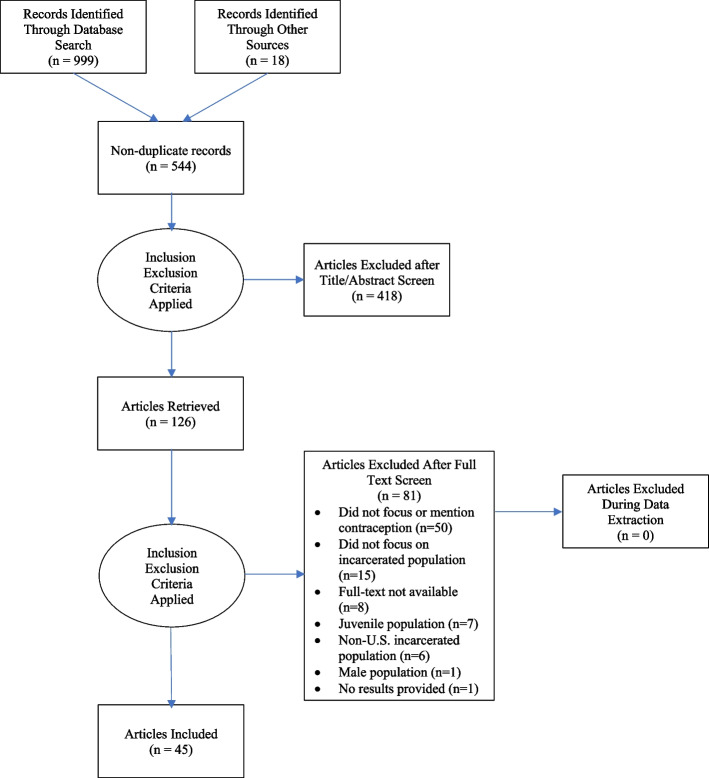
Prisma flowchart here

Figure 1 presents the PRISMA flowchart detailing the inclusion and exclusion of studies. The full search yielded a total of 999 citations. After screening at the abstract and title level and removing duplicate citations, 873 articles were removed. A total of 126 studies received full text appraisal. Ultimately, 81 studies were excluded due to not meeting inclusion/exclusion criteria (see Figure A). A final sample of 45 studies met the inclusion criteria and were included for analysis.

### Methodological quality

Study quality was appraised for the 45 included studies (see Additional file 1). The majority of the included studies were judged to be of good or excellent quality, as indicated by a higher number of yes scores on the critical appraisal tools. A few studies were judged to be “Unclear” on a criterion or two with regard to the Analytical Cross-Sectional and Qualitative appraisal tools because the authors did not explicitly state how they located their research culturally or theoretically or how they addressed the influence of the researcher on the research and vice versa. In seven of the nine studies appraised using the Systematic Review and Research Synthesis tool had items two through nine were marked as “Not Applicable” due to those studies being research synthesis, law reviews, or a simple review describing the state of a topic in a non-systematic review format. Lastly, the remaining two studies were either a scoping or systematic review and were marked “Unclear” because these studies did not directly state that they addressed publication bias and did not report statistical testing pertaining to publication bias, although both included some unpublished grey literature.

### Characteristics of included studies

A comprehensive summary of characteristics for included studies is provided in Table 3. All included studies focused on a United States incarcerated population of women who were of reproductive age. Included studies also provided policy and/or practice recommendations on how to improve contraceptive care for this population. The final sample of studies included 22 cross-sectional, four qualitative, two mixed methods, two systematic reviews, five research syntheses such as a summary on the state of a topic or law review, one randomized controlled trial, and 9 text, position statement, or professional opinion pieces. Studies focused on the effectiveness of contraceptive methods provided to women during incarceration, STI/STD and/or pregnancy prevention, or the ability to provide contraceptive care including access to and continuation of methods, contraceptive counseling, and initial and follow-up medical appointments to address health concerns pertaining to contraception. Studies also focused on the perceptions and experiences of the women receiving contraceptive care while incarcerated, including their access to methods and contraceptive counseling. Most of the studies provided explicit recommendations for improving contraceptive access and care provision to an incarcerated population.

**Table 3:** Summary of study characteristics (*N* = 45)

Study ID	Reference	Type of Study	Location	Methods	Sample	Key Findings
s2	McNeely et al. 2019	Cross Sectional Qualitative	Tennessee	Purposive sampling Interviews	Quantitative = 921 Interviews = 18	Program estimated to have prevented between 270 and 460 unintended pregnancies Family planning information presented at jail info sessions was comprehensive and accurate 18 interviewees felt program was voluntary
s3	Relias Media, 2021*	News Article	Tennessee	N/A	N/A	Family planning information presented at jail info sessions was comprehensive and accurate Incarcerated women did not feel coerced into participating in the program
s4	Sufrin et al. 2015b	Retrospective Descriptive	California	Purposive sampling with secondary data analysis	87 new LARC users	Feasible and safe to provide LARC methods to incarcerated women Correctional facilities should consider increasing access to all available contraceptive methods
s5	Rosengard et al, 2005*	Cross Sectional	Rhode Island	Purposive sampling	221 women aged 18–35	Condom use at last sex, no strong desire to be pregnant, belief that others influence one’s health, and perceived STD risk positively associated women’s intention to use condoms with main partner; pregnancy history negatively associated Condom use at last sex positively associated with women’s intention to use condoms with casual partner; binge drinking and belief that one’s health is largely matter of chance negatively associated
s6	Davis et al. 2018*	Retrospective Descriptive	U.S. Carceral System	Secondary data analysis	U.S. prison population	Medical screenings and reproductive health screenings and services, including contraception services, should be conducted for incarcerated individuals
s14	Sufrin et al. 2009a*	Cross Sectional	U.S. Carceral System	Purposive sampling with surveys	286 correctional health care providers	70% reported some degree of contraceptive counseling but only 11% routinely provided prior to release 70% reported their institution has no formal policy on contraception Only 50% of providers rated their contraceptive counseling ability as good or very good
s16	Sufrin et al. 2015a*	Research Synthesis	U.S. Carceral System	N/A	N/A	Description of access and barriers to accessing sexual and reproductive health care for incarcerated women
s21	Cannon et al. 2018*	Cross Sectional	Illinois	Convenience sampling with surveys	194 women aged 18–50	73.2% of women were at-risk for pregnancy 68% has unprotected sex prior to survey administration 81.4% would be interested in emergency contraception if available 72.7% would be interested in contraceptive supplies if provided free at release
s22	Clarke et al. 2006a*	Cross Sectional	Rhode Island	Purposive sampling with surveys	484 women	84.6% indicated it was likely they would have sex with a male partner within 6 months of release Participants at high risk for STDs and pregnancy, characterized by inconsistent birth control use (66.5%) and condom use (80.4%), multiple partners (38%), and high prevalence of history of unplanned pregnancies (83.6%) and STDs (49%)
s23	Sufrin et al. 2010*	Cross Sectional	California	Purposive sampling with surveys	290 women	71% of all women indicated they would accept an advance supply of emergency contraception upon release from jail 84 women eligible for emergency contraception 68% of all women had misperception about emergency contraception
s24	Oswalt et al. 2010*	Cross Sectional	Southeastern Metropolitan area	Purposive sampling with surveys	188 women of child-bearing age	High rates of STDs, inconsistent contraceptive use, and use of unreliable and user-dependent contraception methods and appeared to need education about contraception methods
s36	Brousseau et al, 2020*	Randomized Controlled Trial	Not Specified	Randomized into control and intervention groups	232 women	Initiation of contraception higher in intervention group, but not significant after controlling number of male partners within 1 year prior to incarceration No significant difference between groups in rates of pregnancies or STDs or continuation of contraception after release
s37	Peart & Knittel, 2020*	Systematic Review	U.S. Carceral System	N/A/	25 studies	Incarcerated women desire access to standard and emergency contraception from carceral health care systems, are concerned about health care providers and manner of care provided, and would like assistance with connecting to community resources
s38	Knittel et al. 2017*	Research Synthesis	U.S. Carceral System	N/A/	N/A	Incarcerated women have distinct health needs from men and provides synthesis of evidence with recommendations for improving reproductive health care for incarcerated women
s39	ACOG, 2021*	Medical Opinion and Position Statement	U.S. Carceral System	N/A	N/A	Recommendations for improving reproductive health care for incarcerated pregnant, postpartum, and nonpregnant women including contraception
s40	Clarke et al. 2006b*	Cross Sectional	Rhode Island	Purposive sampling with interviews	119 women in Phase 1 105 women in Phase 2	Provision of contraception services during incarceration is feasible and greatly increases birth control initiation compared to community-only provision
s41	Sufrin et al. 2017*	Descriptive	Multi-state	N/A/	4 incarcerated contraception service programs	Concerned facilities health administrators, providers, advocates, and legislators should enhance policies for counseling women on family planning and make range of contraception methods available before release
s47	Clarke et al. 2006c*	Cross Sectional	Rhode Island	Convenience sampling with interviews	223 women	Women with negative pregnancy attitudes were significantly more likely to want to start or continue birth control method compared to those with ambivalent pregnancy attitudes
s51	Brousseau et al. 2022*	Cross Sectional	Not Specified	Purposive sampling with surveys	163 women in the community (control group) 141 women in correctional facility (experimental group)	Incarcerated woman less likely to give answer about current or future IUD or implant use Concerns about pain and side effects similar between groups, but incarcerated women more likely to be concerned about device removal and level of training of provider performing device insertion Incarcerated women felt more comfortable with device placement in community than correctional setting
s55	Hayes et al. 2020*	Research Synthesis	U.S. Carceral System	N/A	N/A	By denying access to abortion and contraception, mass incarceration has become a driver of forms of reproductive oppression for people in prison and jails and the community
s56	Smith, 2016*	Opinion	U.S. Carceral System	N/A	N/A	Contraception and contraceptive counseling should be provided in a patient-centered and non-coercive manner
s64	Wenzel et al. 2021*	Cross Sectional	Virginia	Purposive sampling with surveys	95 women at-risk for pregnancy 193 women in total	94% reported vaginal intercourse during 3 months before jail 78% anticipated sex with a man within 6 months of release 47% expressed interest in receiving birth control while jail
s65	Pan et al. 2021*	Cross Sectional	U.S. Carceral System	Convenience sampling with surveys	22 state prison system 6 jails	11 prison and 5 jails permitted permanent female contraception, of which 7 prisons and 3 jails allowed this without a written policy 6 prison and 0 jails provided access to permanent but not reversible contraception
s66	Ravi et al. 2017*	Qualitative	New York	Purposive sampling with interviews	21 women	Trafficking survivors access care for STD and HIV testing, unintended pregnancies, traumas, and chronic diseases Emergency departments, Planned Parenthood, and jails are common care sites Condom use most common form of prevention but inconsistently negotiated due to financial and violent consequences
s71	Ely et al. 2020*	Cross Sectional	Rural Appalachian jails	Secondary data analysis	400 women	96.5% reported lifetime contraception use with 70.5% reporting using multiple methods 69% reported nonuse within last 6 months despite high rates of involvement in risk, intimate male partnership
s72	Hoff et al. 2021*	Systematic Review	U.S. Carceral System	N/A/	28 studies	High rates of contraception underutilization, negative attitudes towards pregnancy, minimal access to reproductive health services including evidence-based contraception, and high rates of unplanned and undesired pregnancies
s78	Ramaswamy et al. 2015*	Longitudinal	Urban Midwestern jail	Purposive sampling with surveys	102 women at baseline 66 women at follow-up	42% of women using highly effective methods prior to incarceration and 54% after release Consistent use of birth control and alcohol problems associated with utilization prior to incarceration and previous pregnancies associated with utilization after release
s79	LaRochelle et al. 2009*	Cross Sectional	California	Purposive sampling with surveys	221 women	61% did not use contraception in past year but 19% of those individuals wanted to This group reported greater difficulty with payment, finding a clinic, and transportation to the clinic relative to those who has used contraception in past year 60% of all women surveyed would accept contraception from Jail Health services if offered
s80	Thompson et al. 2021*	Concurrent Mixed Methods	East Coast Urban jail	Convenience sampling with focus groups and surveys	116 women	In 30 days prior to arrest, 24% using non-barrier contraception method with LARC use being the least 64% not interested in initiating LARC method in jail due to potential LARC side effects and distrust in correctional health care staff’s qualifications Coercion was not listed as a concern
s84	Schonberg et al. 2015*	Qualitative	New York	Semi-structured interviews	32 women	Most participants believed contraception should be provided at jail, but many also said they would be hesitant to use those services Reservations included: negative views of jail health care services, fears about safety of birth control, difficulties associated with follow-up in the community, and desire for pregnancy
s86	Myers et al. 2021	Cross Sectional	Utah	Surveys	148 women aged 18–48	High interest in accessing contraception while in jail Those interested in access during jail more likely to be interested in the injectable, implant, or IUD relative to those who are not interested
s88	Myers, 2018	Qualitative	Utah	Surveys Interviews	194 women 8 jail health care providers	41% of women planned to use contraception after release 67& reported interest in initiating contraception in jail Four providers described comprehensive contraceptive programs in their facilities, 2 providers described limited care, and 2 providers described no contraceptive care available for women
s89	Hunter, 2008*	News Article	New York	N/A	N/A	New York county jails did not have written policies regarding sexual and reproductive or OBGYN care and services Required individuals to quit contraception upon incarceration Reluctance of jail staff to provide care
s91	NCCHC 2020*	Position Statement	U.S. Carceral System	N/A	N/A	Provides recommendations for standards of care and position of the National Commission on Correctional Health Care on correctional health care provision
s92	LaRochelle et al. 2012*	Cross Sectional	California	Surveys	228 reproductive aged women	Difficulty with finding a clinic and transportation to the clinic and payment found in group that had not used contraception in the past year 60% would accept contraception if offered in jail
s93	Hale et al. 2009	Cross Sectional	Southeastern U.S	Surveys	188 women	61.5% did not want to become pregnant, but 76.9% intended to have after release from jail Hight rates of STDs, use of user-dependent and unreliable, and inconsistent use of birth control methods
s94	Sufrin et al. 2012*	Cross Sectional	California	Surveys	9 first year medical students 199 patient visits	Development of medical curriculum for providing OBGYN care for incarcerated patients
s95	Cheedalla & Sufrin, 2021*	Cross Sectional	U.S. Carceral System	Surveys	22 state prisons 6 jails 3 juvenile detention centers	All sites continued use of prescribed method with restrictions on method type and reasons for use 90% of sites allowed individuals to initiate contraception method in custody 65% of sites has formal written contraception policies
s103	Walsh, 2016*	Law Review	New York	N/A	N/A	Poor policies relating to contraception and poor quality of care Patient concerns around access to gynecological exams, sanitary products, and contraception
s118	California Senate Committee on Public Safety 2016	Senate Bill	California	N/A	N/A	Improved access to sanitary or menstruation products, establish wider formulary of contraception methods, and care be provided in non-coercive manner by licensed health care provider
s121	Goodman et al. 2016*	Evidence Review	California	N/A	N/A	Provides recommendations on how to improve contraception access to incarcerated women
s122	Swavola et al. 2016*	Evidence Review	U.S. Jails	N/A	N/A	Despite most incarcerated women interested in beginning contraception either during incarceration or upon release, contraception is not typically available to them
s123	Kraft-Stolar, 2015*	Policy Review	New York	N/A	N/A	Identified problem areas, positive aspects, and recommendations regarding reproductive health care related to severely limited access to contraception for both pregnancy prevention and non-contraceptive benefits
s124	Carey et al. 2008	Research Synthesis	New York	Secondary data analysis Policy review	52 facilities that housed women	No uniform set of policies regarding reproductive health care access No oversight of facilities that create their own policies
s126	Sufrin, 2014*	Qualitative	California	Interviews	40 jail workers, medical staff, and incarcerated women	Jail care can be one of the first contact points for sexual and reproductive health care including contraception Provides recommendations on how to improve for sexual and reproductive health care for incarcerated women

Note: Sufrin et al. 2015a* – Sufrin et al. (2015a*, 2015b)

Sufrin et al. 2015b – Sufrin et.al.  (2015)

Sufrin et al. 2009a*– Sufrin et al. (2009a)*

Clarke et al. 2006a – Clarke et.al,  (2006a)

Clarke et al. 2006b – Clarke et.al.  (2006b)

Clarke et al. 2006c – Clarke et.al.  (2006c)

### Thematic analysis

A total of 49 initial codes emerged across the 45 papers in the final sample. These initial codes, along with their coded segments, were then reexamined and refined by grouping similar themes into overarching categories as well as identifying and combining duplicate codes. A total of 7 overarching themes were identified: 1) policy recommendations, 2) need for contraceptive care, 3) justice agency barriers, 4) policy deficiencies, 5) funding, 6) patients, and 7) health care provider knowledge. Table 4 provides the definitions of these overarching themes and their subthemes and the number of studies, including citations, identified that support each theme and subthemes. Furthermore, exemplar quotes for each theme and subtheme are provided in Additional file 2.

**Table 4:** Definition of themes and subthemes (*N* = 45 studies)

Themes/Subthemes	Definition	Studies Identified Exemplifying Theme/Subtheme
**Policy Recommendations (45)**	Policy recommendations suggested by research, health care providers and organizations	
*Contraception provision during incarceration and prior to release (39)*	Recommendations specifically regarding the provision of contraceptive care to individuals during their incarceration and prior to release from incarceration	s2, s3, s4, s6, s14, s16, s21, s22, s23, s24, s36, s37, s38, s39, s40, s41, s47, s51, s55, s56, s64, s65, s66, s71, s72, s78, s79, s80, s84, s86, s88, s92, s93, s95, s103, s118, s122, s123, s124
*Training and education needs (34)*	Recommendations pertaining to training and educations needs for informing patients, providers, and justice agency personnel about contraception	s2, s4, s5, s14, s16, s21, s23, s37, s38, s39, s40, s41, s51, s55, s64, s65, s71, s72, s78, s80, s84, s86, s88, s89, s91, s94, s95, s103, s118, s121, s122, s123, s124, s126
**Need for Contraceptive Care (32)**	Details the need and benefits of providing contraceptive care to an incarcerated population including the benefits of providing contraception	s2, s3, s4, s5, s6, s16, s21, s22, s23, s36, s37, s38, s40, s41, s47, s55, s64, s65, s66, s71, s72, s78, s79, s80, s84, s86, s88, s92, s93, s123, s124, s126
**Justice Agency Barriers (19)**	Agency barriers inhibiting individuals’ access to contraceptive care during incarceration	
*Reluctance to provide care (11)*	Justice agency unwillingness or hesitancy to provide care or reasoning used to get out of providing care contraceptive care to those who are incarcerated including lack of knowledge and training regarding best medical practices for contraceptive care provision	s2, s14, s37, s41, s71, s86, s88, s95, s103, s123, s124, s126
*Coercive environments, polices, and practices (12)*	Details the restrictive, oppressive, and/or forceful conditions of the carceral environment including its policies, operations, and personnel that strip individuals of their autonomy	s2, s4, s16, s55, s65, s71, s72, s78, s80, s86, s95, s103
**Policy Deficiencies (20)**	Nonexistent, outdated, and/or ambiguous policies that lead to inconsistent or detrimental provision or denial of care	s14, s16, s23, s37, s38, s39, s41, s55, s65, s71, s72, s84, s86, s88, s89, s95, s103, s121, s123, s124
**Funding (15)**	Financial support for contraceptive care provision activities, programs, and supplies	s2, s14, s22, s39, s40, s41, s65, s71, s72, s79, s86, s88, s92, s103, s123
**Patients (22)**	Details the patient perspective, experience, concerns, knowledge, and other patient-related information	
*Patient concerns regarding care (13)*	Patient concerns or questions about the care being provided to them	s37, s38, s51, s64, s66, s71, s72, s80, s84, s86, s103, s123, s126
*Patient knowledge pertaining to contraception (7)*	Patient knowledge about contraception such as, not limited to, factual information, proper use, storage, administration, side effects, health benefits, etc	s2, s3, s80, s84, s92, s93, s123
*Patient desires for contraception during and after incarceration (18)*	Patient indications that they want to start, switch, or stop a contraceptive method during or after incarceration	s21, s22, s23, s37, s38, s41, s47, s64, s66, s71, s72, s80, s84, s86, s88, s92, s123, s126
**Health care Provider Knowledge (17)**	Current provider knowledge and gaps in knowledge or requested trainings, information, and education by providers	s4, s14, s37, s38, s39, s40, s41, s51, s71, s72, s80, s84, s86, s88, s94, s123, s126

Note: Themes are in bold text, subthemes are in italicized text

Note: Numbers in parentheses indicates the number of articles supporting that theme or subtheme. Articles can support more than one theme and/or subtheme

#### Theme 1 – Policy recommendations

Policy recommendations included researchers’ recommendations as well as those attributable to health care providers and organizations. Two types emerged in the reviewed papers: contraception provision during incarceration and prior to release and training and education for justice agency and health care personnel. Table 5 and provides a list of recommendations categorized into these themes, the subthemes within them, and which papers included them. The sections below describe these subthemes.

**Table 5:** Policy recommendations and associated studies

Policy Recommendation	Studies Supporting Policy Recommendation
**Contraception provision during incarceration and prior to release**	
*Provide contraceptive care (e.g., devices, emergency contraception, counseling) (28)*	s2, s3, s4, s6, s14, s16, s21, s23, s36, s37, s38, s39, s40, s41, s47, s64, s65, s71, s,72, s84, s86, s88, s92, s93, s103, s118, s121, s124
*Allow continuation of prior methods (12)*	s14, s37, s39, s41, s55, s65, s71, s84, s86, s88, s118, s123
*Allow initiation, switching, and discontinuing of all methods (15)*	s22, s36, s37, s38, s40, s65, s78, s84, s86, s88, s93, s95, s103, s118, s123
*Provide a comprehensive formulary of methods (12)*	s21, s41, s64, s65, s78, s80, s84, s86, s88, s92, s95, s118
*Provide comprehensive intake screening to assess health risks and needs including emergency contraception and sexual and reproductive health care (9)*	s2, s3, s5, s6, s39, s41, s72, s88, s91
*Establish community connections, justice-health partnerships, and follow-up care (19)*	s2, s3, s4, s37, s38, s39, s41, s51, s65, s66, s78, s79, s80, s84, s86, s92, s95, s103, s123
**Training and education needs**	
*Develop national standard of care including definitions of medically necessary and serious medical need (19)*	s14, s16, s21, s23, s37, s39, s41, s72, s84, s86, s88, s89, s91, s95, s103, s121, s123, s124, s126
*Write formal policies detailing care for facilities (17)*	s14, s16, s37, s38, s41, s65, s71, s72, s84, s86, s88, s89, s95, s103, s121, s123, s124
*Utilize or incorporate a reproductive justice framework (34)*	s5, s14, s16, s21, s22, s23, s24, s36, s37, s38, s40, s41, s47, s55, s64, s65, s66, s71, s72, s78, s84, s86, s88, s89, s91, s92, s93, s103, s118, s121, s122, s123, s124, s126
*Train all staff on legal obligations of care (12)*	s15, s65, s71, s72, s86, s88, s103, s118, s121, s123, s124, s126
*Train all staff on trauma-informed, gender-affirming care (12)*	s16, s37, s39, s72, s86, s88, s89, s91, s103, s121, s123
*Train and provide continuing education, including certifications (14)*	s14, s37, s38, s39, s41, s51, s71, s72, s80, s84, s86, s94, s123, s124
*Train all staff on providing care in a noncoercive manner including how to recognize bias and coercion (19)*	s2, s4, s16, s23, s37, s39, s41, s55, s64, s65, s72, s78, s84, s86, s88, s95, s118, s123, s126
*Train all staff on the benefits of contraception (10)*	s2, s3, s4, s5, s22, s36, s38, s41, s72, s88
*Train all staff on proper records management (6)*	s4, s39, s88, s103, s121, s123

#### Subtheme 1 – Contraception provision

Contraceptive care provision during incarceration and prior to release and improving the continuity of care in the community post-release were identified as one subset of policy recommendations. A large number of included studies indicated incarcerated women should receive contraceptive care to prevent pregnancy and STIs, help treat medical conditions unrelated to pregnancy prevention, and to establish care that many of these women may not have been able to receive prior to incarceration (Myers, 2018*; Myers et al. 2021*; Sufrin et al. 2010* ).^2^ Papers pointed out that jail or prison may be the first contact point for contraceptive care, and thus called for comprehensive intake screening with regard to sexual and reproductive health needs including contraception and emergency contraception (EC; Davis et al. 2018*; Hoff et al. 2021*; McNeely et al. 2019*; Rosengard et al. 2005 ). They call for allowing inmates to continue any current contraceptive methods (ACOG, 2021; Myers, 2018*; Myers et al. 2021*; Sufrin et al. 2009a*) and to start, switch, and/or stop contraceptive methods during their incarceration (Clarke, et al. 2006b; Hale et al. 2009*; Kraft-Stolar, 2015*; Myers et al, 2021*; Pan et al. 2021; Peart & Knittel, 2020*). Authors call for sex education and contraceptive counseling that goes beyond male condoms and provision of prescriptions for a wide formulary of methods (Cannon et al. 2018*; LaRochelle et al, 2012*; Wenzel et al. 2021*). They also call on prison health systems to establish community connections and follow-up care plans and appointments prior to release (Knittel et al. 2017*; McNeely et al. 2019; Ravi et al. 2017*) as the time transitioning back into the community can be fraught with numerous competing priorities such as finding stable housing and employment, avoiding criminal behavior and contacts, reuniting with family, etc. (James, 2014; Makarios et.al. 2010; Visher & Travis, 2011) often identifying obtaining contraception as a lesser priority (Sufrin et al. 2009a*).

#### Subtheme 2 – Training and education needs

Training and education needs were identified for both justice agency and health care personnel. Setting standards for care and developing formal policies were included in this subtheme because the implementation of both depends on training of personnel. Several studies recommended that a standardized set of care requirements and trainings (i.e., legal obligations of care, trauma-informed and gender-affirming care, and continuing education with certifications) could vastly improve contraceptive care provision within the correctional system (Carey et al. 2008*; Cheedalla & Sufrin, 2021*; Sufrin et al. 2009a, 2015a, b). Numerous studies have identified the need to incorporate and utilize a reproductive justice framework, especially when concerning medical and contraceptive care provision. Specifically, justice agency and healthcare personnel must respect an individual’s medical autonomy, including the rights to have children if they desire (e.g., no coerced sterilizations) or to not have children (e.g., access to and continuation of contraceptive methods and abortion services). Other recommendations include training staff, especially justice agency staff, on the administration, benefits, symptoms, and the importance of contraception beyond pregnancy prevention and methods beyond male condoms and oral contraceptive pills (Clarke et al. 2006a*; Sufrin et al. 2017*, 2015a*, b). Lastly, several studies called for training all carceral and health care staff on bias recognition to counteract negative narratives about incarcerated individuals being unfit parents and not entitled to reproductive autonomy (McNeely et al. 2019*; Peart & Knittel, 2020* ).

#### Theme 2 – Need for contraceptive care services

The second theme concerned the need for contraceptive care in a female incarcerated population and the benefits of doing so. Studies identify contraception as a particularly neglected area (Cannon et al. 2018*; McNeely et al. 2019*; Oswalt et al. 2010*; Sufrin et al. 2010, 2017), with incarcerated women having little opportunity to initiate, continue with, or change their chosen method throughout their incarceration (Sufrin, 2014; Sufrin et al. 2009a). Approximately 75% of women are of reproductive age at the time of incarceration (Peart & Knittel, 2020*; Sufrin et al. 2019), with many of these women being at risk for an unintended pregnancy^3^ (Clarke et al. 2006a*; Hale et al. 2009*; Oswalt et al. 2010*) and estimates up to 81% indicating they intended to have sexual relations upon release (Clarke et al. 2006a*; Hale et al. 2009)*. With respect to the non-contraceptive benefits of contraception, papers reference regulating menstruation, decreased risk of some cancers, and treatment of conditions including endometriosis, polycystic ovarian syndrome, and acne (Armstrong, 2010; ACOG, 2010; Jones, 2011). Incarceration may be the first point of contact with sexual and reproductive healthcare, as well as healthcare in general, for many of the women entering the correctional system (Sufrin, 2014; Sufrin et al. 2010). Papers argue that in addition to avoiding unplanned and unwanted pregnancies, STDs/STIs, and the non-contraceptive benefits of contraception, incarcerated patients may benefit from carceral system healthcare as a way to overcome access barriers in the community, which helps individuals focus on other important aspects of reentry (Clarke et al. 2006c*; Hale et al. 2009*; Myers et al. 2021*; McNeely et al. 2019; Oswalt et al. 2010*; Peart & Knittel, 2020*; Rosengard et al. 2005; Sufrin et al. 2017).

#### Theme 3 – Justice agency barriers

The third overarching theme concerned the barriers inhibiting individuals’ access to contraceptive care during incarceration. This theme was identified by its two subthemes: 1) reluctance to provide care, and 2) coercive environment and practices.

#### Subtheme 1 – Reluctance to provide care

While justice agencies are required to provide medical care to incarcerated individuals, the definitions of adequate, necessary, and serious medical care have been vaguely defined and primarily left up to the agencies to define (Carey et al. 2008*), which may lead to a reluctance to provide contraceptive care. Various studies point out that nonmedical justice agency personnel do not receive training and education regarding medical situations, prescriptions, or the need, benefits, or harms for prescriptions, and that knowledge is particularly scant as it relates to contraception (Ely et al. 2020; Kraft-Stolar, 2015* ; Sufrin et al. 2017). Interpretations of medical situations, severity of issues or need, and care are left to nonmedical, or nonmedically trained, personnel. Studies described such reluctance as based in the belief that contraceptive care is not medically necessary (i.e., understanding reasons why women might need to access or use contraception while incarcerated), belief that incarcerated women do not engage in potentially procreative sex, concerns about costs, and the claim that contraceptive care is outside their responsibilities (Cheedalla & Sufrin, 2021*; Sufrin et al. 2017). These narrow views of contraceptive care belie the facts regarding the benefits of providing care and can have disastrous health consequences for incarcerated women in the future, putting them at risk for hormonal imbalance and unwanted pregnancy (Hunter, 2008*; Myers, 2018*; Walsh, 2016*).

#### Subtheme 2 – Coercive environment and practices

Some studies detail the restrictive, oppressive, and/or forceful conditions of the carceral environment including its policies, operations, and personnel that strip individuals of their autonomy. These studies emphasize that incarcerated individuals are, in many ways, at the mercy of the administrators and line officers and dependent on them for numerous things such as access to care, commissary, and group activities, and that officers have broad latitude to exact punishment for actual or perceived transgressions (Kraft-Stolar, 2015). The current environment in most facilities is not designed to provide quality health care, nor is it designed to allow the freedom of choice necessary to seek medical care, make medical decisions that can benefit the patient, or to safely, swiftly, and effectively navigate the ever-changing needs of medical care (Myers, 2018*; Sufrin, 2014; Sufrin et al, 2015a, 2015b).

#### Theme 4 – Policy deficiencies

The policy deficiencies the papers in the sample pointed out included nonexistent, outdated, and/or ambiguous policies that lead to inconsistent or detrimental provision or denial of care. Many policies are outdated and not in line with contemporary understandings of best practice standards for health care provision (Cheedala & Sufrin, 2021; Hoff et al. 2021*; Kraft-Stolar, 2015*). Additionally, a surprising number of systems lack policies related to contraceptive health care provision (Sufrin et al. 2009a). Sufrin and colleagues (2015a) found facility staff have broad latitude to determine what constitutes a serious medical need. Pan and colleagues (2021) found a small number of institutions that allow contraception use or patients to obtain permanent contraception without a formal policy in place. While this is better than a policy denying incarcerated people needed care, without a formal policy to provide contraception care it could be denied at any time.

#### Theme 5 – Funding

Funding, which consists of financial support for contraceptive care provision activities, programs, and supplies, was the fifth theme to emerge. Without funding, services may be denied even with policies guaranteeing care provision. Justice agencies and health care providers must make due with limited resources to provide the constitutionally required care, as well as specialty care, and maintain the medical staff adequate to care for the number of incarcerated patients (Kraft-Stolar, 2015*; Sufrin, 2014). However, some studies emphasized the potential cost savings, via cost avoidance, that contraceptive care provision to those who are incarcerated could generate. Two studies introduce the model of justice-health center partnerships as a way to control the cost of providing contraceptive care (McNeely et al. 2019; Sufrin et al, 2017). Contraception provision also can help avoid the expense of transportation for pregnant inmates to health care or abortion appointments and avoid lawsuits for the denial of care (Sufrin, 2014). One study found that the U.S. government saved $7.09 for every dollar spent on contraception (Frost et al. 2014), and suggested that similar savings would take place in carceral settings. Given the vast potential for benefits, it makes sense from an economic perspective for both health care organizations and justice agencies to provide contraceptive care to those who are incarcerated.

#### Theme 6 – Patients

The sixth overarching theme includes patient perspective, experience, concerns, knowledge, and other patient-related information. This three has three subthemes: 1) patient concerns, 2) patient knowledge pertaining to contraception, and 3) patients’ desire for contraception.

#### Subtheme 1 – Patient concerns

Patient concerns described in the selected papers referred to doubts about provider knowledge about contraception, bedside manner, quality of care received, low trust of medical staff, concern about contraceptive method side effects, access to contraception and follow-up care, and stigma for wanting or using contraception while incarcerated (Hoff et al. 2021*; Kraft-Stolar, 2015*; Peart & Knittel, 2020*; Schonberg et al. 2015*; Thompson et al. 2021*). Papers described patients who want to feel like their providers hear their concerns and work together with them to find the best available option to treat their medical needs, but many did not feel their provided did this (Brousseau et al. 2022*; Kraft-Stolar, 2015*; Peart & Knittel, 2020*). Health care providers can forge a connection or bond with their patients to help alleviate their concerns and are in a position to provide more than medical care to patients in an otherwise dismal time (Kraft-Stolar, 2015*). These concerns were present in institutions across the country. These findings suggest there is much work to be done to improve patients’ experiences of seeking and receiving care while incarcerated and to improve the experiences and likelihood of seeking future medical care.

#### Subtheme 2 – Patient knowledge pertaining to contraception

Studies identified that women have misconceptions about EC and proper contraceptive use (Cannon et al. 2018*; Sufrin et al. 2010). In another study very few incarcerated women accurately described potential side effects, how long a long-acting reversible contraceptive (LARC) method can stay in place, or knew that they could return to any health department upon release to address complications or have their LARC removed (McNeely et al. 2019). These findings demonstrate a need for contraceptive care and education programming within the carceral system that encompasses proper use, storage, administration, side effects, health benefits and works to combat misinformation and misperceptions. Failure to address these misperceptions pertaining to contraception can lead to women not utilizing contraception in the future, thus putting them at risk for an unplanned or unwanted pregnancy.

#### Subtheme 3 – Patient desire for contraception

Studies generally reported that patients wanted to start, switch, or stop a contraceptive method during or after incarceration and that they desired connections to providers of contraceptive care post-release (Cannon et al. 2018*; Myers, 2018*; Myers et al. 2021*; Peart & Knittel, 2020*). Several studies found that patients were very likely to accept EC or contraception prescription prior to leaving jail (Cannon et al. 2018*; Clarke et al. 2006a*, 2006c; LaRochelle et al. 2012*; Schonberg et al. 2015*; Sufrin et al. 2010). Patients desired contraception for several reasons such as a desire to prevent future pregnancy (Clarke et al. 2006c*; Gutierres & Barr, 2003; Hoff et al. 2021*; Thompson et al. 2021*) and because they did not know how to or if they could access contraception in the community (Hale et al. 2009*; Peart & Knittel, 2020*; Schonberg et al. 2015*).

#### Theme 7 – Health care provider knowledge

Studies addressing health care provider knowledge found that levels of knowledge among health care providers who work with a justice-involved population vary significantly. Some providers and programs provide comprehensive and accurate contraceptive care (see Sufrin et al. 2017 for program examples). However, a sizeable portion of providers have noted that they would benefit from additional education about contraception (Sufrin et al. 2009a). This suggests clinicians want to provide quality care to incarcerated individuals but may lack the knowledge to do so.

## Discussion

The seven themes identified by this review shed light on the gaps, barriers, and facilitators of current contraceptive service provision to those who are incarcerated. Key issues identified were: 1. the clear need for contraceptive services to be provided to those who are incarcerated, 2. lack of clear standards and policy pertaining to contraception, as well as sexual and reproductive health in general, 3. justice agency personnel and health care provider education and training needs, including bias recognition, 4. patient knowledge about and desire for contraception during and after incarceration and the concerns about the care they receive, 5. potential funding sources that justice agencies and health care providers can utilize to help finance contraceptive care in addition to the medical care already being provided, and 6. several policy recommendations that could address the issues of and improve the current state of contraceptive care provision.

Our findings accomplished two important goals of NIRN’s Exploration phase. First, the compiled evidence in this systematic review identifies a clear need for change regarding policies and practices pertaining to contraceptive care service provision to incarcerated women in the United States. For example, 20 studies found policy deficiencies within carceral facilities across the United States that were outdated and not in line current best practices or no official or formal policy pertaining to contraceptive care during incarceration. Additionally, 12 studies identified a hesitancy or reluctance to provide care due to lack of staff education and knowledge pertaining to contraception. Lastly, 32 studies identified a need for contraceptive care services to be provided during incarceration, preparation for release, and to connect individuals to services post-release. These are the most glaring issues identified in the literature by the systematic review though other issues were present.

Second, our findings identified several evidence-based solutions supported by both research and professional health care organizations to address the identified need for change. For example, to address policy deficiencies and lack of standards of care, studies have recommended the development of national standards of care for contraceptive care service provision and developing or updating of formal policies detailing care in carceral facilities. Also, providing training and education to both justice agency and health care to bolster staff knowledge pertaining to contraception use, benefits, and problems can address hesitancy and reluctance to provide or allow the continued use of contraception to individuals during incarceration. This systematic review has identified numerous other policy and practice recommendations designed to improve contraceptive care service provision during incarceration.

The remaining aspects of the Exploration phase need to be completed by agencies wanting to change their current practices or adopt new practices. Agencies will need to develop an implementation team and select champions to spearhead those teams and develop communication processes to support the work to move through the implementation process. Furthermore, these champions will need to assess the level of readiness for change within the organization and identify barriers and facilitators that can hinder and help the implementation process. While this systematic review provides part of the groundwork of identifying the needs of and evidence-based solutions for improving contraceptive care provision to justice-involved women, ultimately, the decision to change current or adopt new policies and practices lies with the agency.

### Limitations

There are three limitations with this research. First, the search might not have identified all relevant materials. Justice agency in-house memos, documents, and policies and unpublished technical reports, including those that did not receive permission to be published or shared outside of the agency, were not included in the systematic review. There potentially could be critical information in those unavailable materials that could help refine our themes and subthemes. Second, this review focused only on those who identify as and are biologically female as the majority of contraceptive methods are designed for those who are biologically female. Future studies should examine contraceptive care provision to those who are biologically and identify as male and trans in order to identify, develop, and implement more comprehensive and gender-affirming contraceptive care to those who are incarcerated. Lastly, despite gathering materials that have studied locations across the United States including two national surveys of justice agencies, results may not be generalizable to all carceral facilities in the United States. With so few formal programs or services in place (see Sufrin et al. 2017 for examples), program evaluations or descriptions, and the limited number of formal policies, it is difficult to grasp the true reality of how contraceptive services are provided at the granular or individual agency level.

## Conclusion

Contraception is an important aspect of healthcare and there is a clear need for improved healthcare for individuals involved in the justice setting. Given the potential for coercion and abuse implementation of these programs must be approached through a person-centered lens to ensure autonomy and informed consent. However, with all of these cautions in place there is a clear need for contraceptive services for women involved with the correctional system. As several researchers (Myers, 2018*; Myers et al. 2021*; Sufrin et al. 2010) have mentioned, the correctional system may be the first point of contact for sexual and reproductive healthcare, as well as general healthcare, for many women to get the care they want but may have been unable to get due to a variety of barriers. However, given the history of atrocities when providing contraceptive and sexual and reproductive health care to incarcerated women (see Ross & Solinger, 2017), as well as recent events such as California’s and Tennessee’s coercive use of sterilization (Hawkins, 2017; Kouros, 2013; Roth & Ainsworth, 2015; Winters & McLaughlin, 2020), contraceptive care provision must be conducted in a patient-centered manner without bias or coercion. Furthermore, both justice agency and health care personnel would benefit from educational and training sessions to better understand the necessity and benefits of contraceptive care. The correctional system is in a unique position to deliver much-needed care, which would result in many potential benefits to the individuals, the correctional system, and the community at large.

### Supplementary Information

**Additional file 1. **Study Quality Appraisals.

**Additional file 2. **Exemplar Quotes for Identified Themes and Subthemes.

## Data Availability

All data generated or analyzed during this study are included in this published article and its supplementary information files.

## Footnotes

1 We acknowledge that not all individuals who have the capacity to become pregnant or deliver a baby identify as women and that not all pregnancies result from couples who identify as heterosexual. Trans men, gender expansive individuals, and individuals in queer relationships may experience pregnancy. We use the term women to broadly discuss the biological and gendered experience of pregnancy and female-based contraception methods for individuals with the capacity to become pregnant.

2 See section Theme 2 – Need for Contraceptive Care for further discussion.

3 Individuals could be at-risk for an unintended pregnancy for varying reasons such as they do not wish to become pregnant, are not using a contraceptive method, did not know if they could access or how to access contraception in the community, and/or plan to be sexually active after release from incarceration.
