# Supplementary material for: Building an implementation framework to address unmet contraceptive care needs in a carceral setting: a systematic review
Source: Health Justice. 2023 Oct 20;11:43. doi: 10.1186/s40352-023-00243-8 (PMC10588068; doi:10.1186/s40352-023-00243-8)
Supplement: Supplementary file 1 — Additional file 1. Study Quality Appraisals. [file 40352_2023_243_MOESM1_ESM.docx]

Appendix 1 – Study Quality Appraisals

JBI Study Appraisal Analytical Cross-Sectional Tool

| Record Information | Q1 | Q2 | Q3 | Q4 | Q5 | Q6 | Q7 | Q8 |
| --- | --- | --- | --- | --- | --- | --- | --- | --- |
| s2 | Yes | Yes | Yes | Yes | Unclear | Unclear | Yes | Yes |
| s4 | Yes | Yes | Yes | Yes | Yes | Yes | Yes | Yes |
| s5 | Yes | Yes | Yes | Yes | Yes | Yes | Yes | Yes |
| s14 | Yes | Yes | Yes | Yes | Yes | Yes | Yes | Yes |
| s21 | Yes | Yes | Yes | Yes | Yes | Yes | Yes | Yes |
| s22 | Yes | Yes | Yes | Yes | Yes | Yes | Yes | Yes |
| s23 | Yes | Yes | Yes | Yes | Yes | Yes | Yes | Yes |
| s24 | Yes | Yes | Yes | Yes | Yes | Yes | Yes | Yes |
| s25 | Yes | Yes | Yes | Yes | Yes | Yes | Yes | Yes |
| s31 | Yes | Yes | Yes | Yes | Yes | Yes | Yes | Yes |
| s40 | Yes | Yes | Yes | Yes | Yes | Yes | Yes | Yes |
| s46* | -- | -- | -- | -- | -- | -- | -- | -- |
| s47 | Yes | Yes | Yes | Yes | Yes | Yes | Yes | Yes |
| s51 | Yes | Yes | Yes | Yes | Yes | Yes | Yes | Yes |
| s64 | Yes | Yes | Yes | Yes | Yes | Yes | Yes | Yes |
| s65 | Yes | Yes | Yes | Yes | Yes | Yes | Yes | Yes |
| s71 | Yes | Yes | Yes | Yes | Yes | Yes | Yes | Yes |
| s78 | Yes | Yes | Yes | Yes | Yes | Yes | Yes | Yes |
| s79* | -- | -- | -- | -- | -- | -- | -- | -- |
| s85 | Yes | Yes | Yes | Yes | Yes | Yes | Yes | Yes |
| s86 | Yes | Yes | Yes | Yes | Yes | Yes | Yes | Yes |
| s88 | Yes | Yes | Yes | Yes | Yes | Yes | Yes | Yes |
| s92 | Yes | Yes | Yes | Yes | Yes | Yes | Yes | Yes |
| s93 | Yes | Yes | Yes | Yes | Yes | Yes | Yes | Yes |
| s94 | Yes | Yes | Yes | Yes | Yes | Yes | Yes | Yes |
| s95 | Yes | Yes | Yes | Yes | Yes | Yes | Yes | Yes |
| s122 | Yes | Yes | Yes | Yes | Unclear | Unclear | Yes | Yes |
| s123 | Yes | Yes | Yes | Yes | Unclear | Unclear | Yes | Yes |

Note: *article was a poster abstract that met the inclusion criteria but could not be appraised given current information

Q1: Were the criteria for inclusion in the sample clearly defined?

Q2: Were the study subjects and the setting described in detail?

Q3: Was the exposure measured in a valid and reliable way?

Q4: Were objective, standard criteria used for measurement of the condition?

Q5: Were confounding factors identified?

Q6: Were strategies to deal with confounding factors stated?

Q7: Were the outcomes measured in a valid and reliable way?

Q8: Was appropriate statistical analysis used?

JBI Study Appraisal Qualitative Research Tool

| Record Identification | Q1 | Q2 | Q3 | Q4 | Q5 | Q6 | Q7 | Q8 | Q9 | Q10 |
| --- | --- | --- | --- | --- | --- | --- | --- | --- | --- | --- |
| s2 | Yes | Yes | Yes | Yes | Yes | Unclear | No | Yes | Yes | Yes |
| s31 | Yes | Yes | Yes | Yes | Yes | Unclear | Unclear | Yes | Yes | Yes |
| s66 | Yes | Yes | Yes | Yes | Yes | Unclear | Unclear | Yes | Yes | Yes |
| s74 | Yes | Yes | Yes | Yes | Yes | Unclear | Unclear | Yes | Yes | Yes |
| s80 | Yes | Yes | Yes | Yes | Yes | Unclear | Unclear | Yes | Yes | Yes |
| s84 | Yes | Yes | Yes | Yes | Yes | Unclear | Unclear | Yes | Yes | Yes |
| s88 | Yes | Yes | Yes | Yes | Yes | Unclear | Unclear | Yes | Yes | Yes |
| s96 | Yes | Yes | Yes | Yes | Yes | Unclear | Unclear | Yes | Yes | Yes |
| s126 | Yes | Yes | Yes | Yes | Yes | Unclear | Unclear | Yes | Yes | Yes |

Note:

Q1: Is there congruity between the stated philosophical perspective and the research methodology?

Q2: Is there congruity between the research methodology and the research question or objectives?

Q3: Is there congruity between the research methodology and the methods used to collect the data?

Q4: Is there congruity between the research methodology and the representation and analysis of data?

Q5: Is there congruity between the research methodology and the interpretation of the results?

Q6: Is there a statement locating the researcher culturally or theoretically?

Q7: Is the influence of the researcher on the research, and vice-versa addressed?

Q8: Are participants, and their voices, adequately represented?

Q9: Is the research ethical according to current criteria or, for recent studies, and is there evidence of ethical approval by an appropriate body?

Q10: Do the conclusions drawn in the research report flow from the analysis, or interpretation, of the data?

JBI Study Appraisal Systematic Review and Research Synthesis Tool

| Record Identification | Q1 | Q2 | Q3 | Q4 | Q5 | Q6 | Q7 | Q8 | Q9 | Q10 | Q11 |
| --- | --- | --- | --- | --- | --- | --- | --- | --- | --- | --- | --- |
| s16* | Yes | NA | NA | NA | NA | NA | NA | NA | NA | Yes | Yes |
| s37 | Yes | Yes | Yes | Yes | Yes | Yes | Yes | Yes | Unclear | Yes | Yes |
| s38* | Yes | NA | NA | NA | NA | NA | NA | NA | NA | Yes | Yes |
| s41* | Yes | NA | NA | NA | NA | NA | NA | NA | NA | Yes | Yes |
| s72 | Yes | Yes | Yes | Yes | Yes | Yes | Yes | Yes | Unclear | Yes | Yes |
| s73* | Yes | NA | NA | NA | NA | NA | NA | NA | NA | Yes | Yes |
| s103* | Yes | NA | NA | NA | NA | NA | NA | NA | NA | Yes | Yes |
| s104* | Yes | NA | NA | NA | NA | NA | NA | NA | NA | Yes | Yes |
| s121* | Yes | NA | NA | NA | NA | NA | NA | NA | NA | Yes | Yes |

Note* Not applicable (NA) responses were because the article was either a law review, research synthesis, or utilizing research to summarize the state of findings

Q1: Is the review question clearly and explicitly stated?

Q2: Were the inclusion criteria appropriate for the review question?

Q3: Was the search strategy appropriate?

Q4: Were the sources and resources used to search for studies adequate?

Q5: Were the criteria for appraising studies appropriate?

Q6: Was critical appraisal conducted by two or more reviewers independently?

Q7: Were there methods to minimize errors in the data extraction?

Q8: Were the methods used to combine studies appropriate?

Q9: Was the likelihood of publication bias assessed?

Q10: Were recommendations for policy and/or practice supported by the reported data?

Q11: Were the specific directives for new research appropriate?

JBI Study Appraisal Randomized Controlled Trials Tool

| Record Identification | Q1 | Q2 | Q3 | Q4 | Q5 | Q6 | Q7 | Q8 | Q9 | Q10 | Q11 | Q12 | Q13 |
| --- | --- | --- | --- | --- | --- | --- | --- | --- | --- | --- | --- | --- | --- |
| s36 | Yes | Yes | Yes | Yes | Unclear | Unclear | Yes | Yes | Yes | Yes | Yes | Yes | Yes |

Note:

Q1: Was true randomization used for assignment of participants to treatment groups?

Q2: Was allocation to treatment groups concealed?

Q3: Were treatment groups similar at baseline?

Q4: Were participants blind to treatment assignments?

Q5: Were those delivering treatment blind to treatment assignment?

Q6: Were outcomes assessors blind to treatment assignment?

Q7: Were treatment groups treated identically other than the intervention of interest?

Q8: Was follow up complete if not, were differences between groups in terms of their follow up adequately described and analyzed?

Q9: Were participants analyzed in the groups to which they were randomized?

Q10: Were outcomes measured in the same way for treatment groups?

Q11: Were outcomes measured in a reliable way?

Q12: Was appropriate statistical analysis used?

Q13: Was the trial design appropriate, and any deviations from the standard RCT designed (evaluation, randomization, parallel groups) accounted for in the conduct and analysis of the trials?

JBI Study Appraisal Text and Opinion Tool

| Record Identification | Q1 | Q2 | Q3 | Q4 | Q5 | Q6 |
| --- | --- | --- | --- | --- | --- | --- |
| s3 | Yes | Yes | Yes | Yes | Yes | Yes |
| s6 | Unclear | Yes | Yes | Yes | Yes | Yes |
| s39 | Yes | Yes | Yes | Yes | Yes | Yes |
| s55 | Yes | Yes | Yes | Yes | Yes | Yes |
| s56 | Yes | Yes | Yes | Yes | Yes | Yes |
| s67 | Yes | Yes | Yes | Yes | Yes | Yes |
| s89 | Yes | Yes | Yes | Yes | Yes | Yes |
| s91 | Yes | Yes | Yes | Yes | Yes | Yes |
| s118 | Yes | Yes | Yes | Yes | Yes | Yes |
| s120 | Yes | Yes | Yes | Yes | Yes | Yes |
| s124 | Yes | Yes | Yes | Yes | Yes | Yes |

Note:

Q1: Is the source of the opinion clearly identified?

Q2: Does the source of the opinion have standing in the field of expertise?

Q3: Are the interests of the relevant populations the central focus of the opinion?

Q4: Is the stated position the result of an analytical process, and is there logic in the opinion expressed?

Q5: Is there reference to the extant literature?

Q6: Is any incongruence with the literature/sources logically defended?
