# Supplementary material for: Building an implementation framework to address unmet contraceptive care needs in a carceral setting: a systematic review
Source: Health Justice. 2023 Oct 20;11:43. doi: 10.1186/s40352-023-00243-8 (PMC10588068; doi:10.1186/s40352-023-00243-8)
Supplement: Supplementary file 2 — Additional file 2. Exemplar Quotes for Identified Themes and Subthemes. [file 40352_2023_243_MOESM2_ESM.docx]

Appendix 2 – Exemplar Quotes for Identified Themes and Subthemes

| **Theme/Subtheme** | **Exemplar Quotes** |
| --- | --- |
| **Policy Recommendations** |  |
| *Contraception provision during incarceration and prior to release* | Sufrin et al (2015a) – Disruptions in contraceptive use have significant implications for women in jail, who are often there for short periods of time; a lapse in use of a hormonal method puts women at risk for unintended pregnancy when they get released. |
|  | Sufrin et al (2017) – Upon entry into the booking jail, women are screened for eligibility for emergency contraception, and referred to a clinician. OCPs [oral contraceptive pills] and DMPA [Depo-Provera shot] are currently on the jail’s routine formulary, and pills are prescribed as a self-carry medication. Prescriptions for [birth control] pills or written reminders of when the next DMPA inject is due are placed in a woman’s property for release, along with information on local pharmacies and clinics. Prescriptions and information are also provided if a woman wants to initiate the contraceptive patch or ring. |
|  | Schonberg et al (2015) – A few women expressed that offering birth control at the jail was pointless because of barriers to follow-up care. One woman said, “If there’s a facility that some women can go to follow-up on the birth control, that would be great. But if they can’t, then what’s the point? You’re setting them up for a fall. You’re going to give them birth control while they’re in jail with a bunch of other women, and it’s not going to work when they’re outside in the real world with the men.” Concerns about follow-up were especially prevalent regarding long-acting reversible contraception (LARC), like the intrauterine device (IUD) or a subdermal contraceptive implant. One woman said, “Really, what do you do if you get something in here, and there’s no facility outside to help you with the aftercare? Now you’re stuck.” |
| *Training and education needs* | Sufrin (2014) – Although the NCCHC is not a legal organization, the juridical framework established by *Estelle* nonetheless gets played out through the organization’s regulatory guidance for prison and jail clinics. Standardizing clinical care offers jails and prisons a road map for avoiding deliberate indifference, for providing what the NCCHC deems a “constitutionally acceptable level of care” (NCCHC website). I met a jail nursing administrator from Arkansas at NCCHC’s annual conference who told me that her facility’s accreditation has saved them from several lawsuits. “It’s an assurance,” she told me, “That you’re not showing ‘deliberate indifference.’” She rolled her eyes with disdain as she uttered those constitutionally bound words. The legal mandate was imbricated into her sense of professional obligation. |
|  | Goodman et al (2016) – Effectively implement policies, with training, monitoring and accountability. The best written policy means nothing if jail and medical staff do not know about the policy, are not trained in the policy, compliance with the policy is not monitored, and there is no accountability for failing to adhere to the policy. Once jails adopt new policies they must devote energy to training, periodic compliance monitoring, and addressing implementation problems. Jails cannot turn a blind eye if policies are ignored and routinely flouted. |
| **Need for Contraceptive Care Services** | Sufrin et al (2015b) – In addition to being at high risk for unintended pregnancies, incarcerated women have high rates of STDs and are thus at risk for pelvic inflammatory disease. |
|  | Knittel et al (2017) – Incarcerated women could also benefit from the non-contraceptive benefits of many contraceptive methods, including menstrual regulation, decreased risk of some cancers, and treatment of medical conditions such as polycystic ovarian syndrome, acne, endometriosis, fibroids, and pelvic pain. |
|  | Sufrin et al (2015b) – The incarcerated women in our study were able to access highly effective contraceptives, which they might not have been able to do in their communities. |
| **Justice Agency Barriers** |  |
| *Reluctance to provide care* | McNeeley et al (2019) – The county jails declined to participate in the program if it included screening for STIs or cervical cancer. The sheriffs were concerned that their health care costs would increase unsustainably. |
|  | Cheedalla & Sufrin (2021) – In many facilities, women could not continue contraception because it was not considered a “medical need,” despite its emphasis by national organizations such as NCCHC and ACOG. Contraception may not be considered medically necessary by institutions because of the decreased likelihood of sexual intercourse during incarceration. |
|  | Sufrin et al (2017) – Various administrators and staff initially expressed resistance to providing contraception during incarceration because of the potential suggestion that women were at risk for pregnancy while in custody and because they believed “It is not our problem.” |
| *Coercive environments, polices, and practices* | McNeeley et al (2019) – The prison guards play an important role… and jails are coercive environments and the guards hold the power. |
|  | Smith (2016) – The power differential between and provider in a prison setting further exacerbates this concern. |
|  | Sufrin et al (2015b) – Jail and prison are inherently coercive settings in which individuals’ autonomy is deliberately restricted. Furthermore, incarcerated persons may fear punishment for following what they perceive to be instructions from an authority figure, such as a clinician. |
| *Personnel training and education needs* | Sufrin et al (2009a) – There also appears to be a need for improved contraception education for correctional health providers, and most of our respondents were eager for this knowledge. Although the most commonly counseled, prescribed and dispensed method was oral contraceptive pills (OCPs), only 50% of clinicians indicated that they felt “good” or “very good” in their OCP counseling abilities. This lack of confidence in OCP knowledge is also disheartening, since more than half of respondents were specialists in fields that should know about OCPs: family medicine, obstetrics and gynecology, internal medicine, and pediatrics. |
|  | Walsh (2016) – DOCCS [in New York] appears to recognize few specific instances in which women can access limited forms of contraception. While DOCCS policy does allow for some women to access contraception in the specific instances…, these policies fail to account for the variety of reasons incarcerated women may want access to prescription contraception. Furthermore, interviews with officials at correctional facilities revealed a lack of understanding around the reasons a woman might need to access contraception while incarcerated. |
| **Policy Deficiencies** | Cheedala & Sufrin (2021) – The variability in contraception policies across prisons, jails, and juvenile detention systems evidenced in this study speaks of the reality for incarcerated women—that their reproductive well-being is largely dependent on institutions of incarceration, and on which one they are in. It is important to advocate for standardized policies across prisons and jails that allow women to use contraception for contraceptive and noncontraceptive reasons to sufficiently meet their reproductive health needs. |
|  | Walsh (2016) – Inconsistent policies make it difficult for most women to obtain contraception while incarcerated. |
|  | Sufrin et al (2009a) – Lack of uniform policy surrounding contraception for incarcerated women. 70% said their institution had no formal policy and 11% were unsure if their facility had a policy. |
| **Funding** | Myers (2018) – Women in jail are representative of a medically underserved population and often lack health insurance and struggle to access or afford reproductive health care and effective methods of contraception in the community. |
|  | McNeeley et al (2019) – The major challenges to program implementation stemmed from the combination of high rates of poverty and the lack of public financing for health care in East Tennessee. |
|  | Sufrin et al (2009a) – Due to the high rates of recidivism coupled with the costs of prenatal care or abortion services for pregnant inmates, making birth control available while women are still in jail or prison would likely reduce health care costs overall. |
|  | Myers (2018) – The cost savings generally come from helping women avoid unwanted pregnancies that would have gone on to require publicly funded pregnancy-related care. |
| **Patients** |  |
| *Patient concerns regarding care* | Peart & Knittel (2020) – Women across multiple studies reported concern that providers were either not knowledgeable about contraceptive options or would seldom discuss side effects… or being cared for by medical trainees. |
|  | Hoff et al (2021) – Despite generally positive attitudes toward contraception provision for WICJ [women in the criminal justice system], some expressed concerns about accessing contraception services during incarcerated related to the following: misperceptions about EC being an abortifacient, stigma of using contraception in jail, and general mistrust of the medical community. WICJ experiences high levels of mistrust of medical care in [criminal justice] facilities, misperceptions and lack of education surrounding contraceptive option, and concerns about follow-up care. |
| *Patient knowledge pertaining to contraception* | Sufrin et al (2010) – 68% had a misperception about EC causing an abortion, being unsafe, or it being ineffective. Less than one quarter (23%) were aware that EC is available over the counter, and only 5% could correctly identify that EC can be take up to 5 days after unprotected sex. |
|  | Oswalt et al (2010) – Women leaving jail or prison appear to be an ideal population for providing education on safe sex and proper condom use. |
|  | Hale et al (2009) – Many women may be uncertain as to how obtain contraceptives or how to use them; having a nurse educator explain them the basics of contraceptive option and instructions as to how to use them correctly could greatly benefit such women. |
| *Patient desires for contraception during and after incarceration* | Cannon et al (2018) – 73% of respondents were interested in contraception supplied if provided free of charge just prior to release and 82% of women were interested in receiving a free supply of emergency contraception. |
|  | LaRochelle et al (2012) – 60% of all women surveyed in San Francisco desired contraception be available through the jail health services and would accepts its use if offered. |
| **Health care Provider Knowledge** | McNeeley et al (2019) – The information delivered in the comprehensive family planning education sessions was accurate and nurses informed the women in the education sessions and during clinic visits about the specifics of getting follow-up care in the particular county where they worked. |
|  | Sufrin et al (2009a) – Although the most commonly counseled, prescribed, and dispended method was oral contraceptive pills (OCPs), only 50% of clinicians indicated that they felt ‘good’ or ‘very good’ in their OCP counseling abilities. There also appears to be a need for improved contraception education for correctional health providers, and most our respondents were eager for this knowledge… with 84% of clinicians felt they themselves would benefit from additional education about contraception. |
